# Supplementary material for: Dependence of learning outcomes in flipped and lecture classrooms on review questions: A randomized controlled trial and observational study
Source: PLoS One. 2022 Dec 19;17(12):e0279296. doi: 10.1371/journal.pone.0279296 (PMC9762591; doi:10.1371/journal.pone.0279296)
Supplement: Attachment — Submitted filename: PLoS ONE Flipped Class Manuscript - Response to Reviewers.docx [file pone.0279296.s001.docx]

November 28, 2022

Dear Editors and Reviewers,

Thank you for your comments on our manuscript. Below, we list each comment, our response, and summarize any changes made to the manuscript. To improve readability, we addressed comments from each source on separate pages (i.e., academic editor, journal requirements, reviewer 1, & reviewer 2). We addressed all comments to the best of our understanding and ability. The revised manuscript is much stronger as a result.

Below is a summary of all changes made to the manuscript and associated files.

- Minimal anonymized datasets were uploaded to <https://osf.io/qw4x9/>.
- Figures were renamed to match PLOS ONE style requirements.
- The secondary analysis in Study 2 was described in the Introduction.
- “IRB Statement” was renamed to “Ethics Statement” and describes informed consent better. The IRB letter of exemption was uploaded as an attachment.
- Subheadings were added to the Results and Discussion sections.
- The Discussion section was rewritten to better describe the provided citations and their relation to our work.

Thank you for considering our manuscript for publication. We appreciate your time and look forward to your response.

Sincerely,

Jason Pitt, PhD	Bethany Huebner, PT, DPT, PhD

Assistant Professor	Associate Professor

# Comments from the Academic Editor

## Comment: “As the study is randomized controlled trial, authors are requested to comment on Trial Registry & Institutional Ethical Committee approval.”

| **Response** | **Changes made** |
| --- | --- |
| We designed our study as a randomized controlled trial in order to maximize internal validity. Because this was not a clinical trial, we did not pre-register the study. This is consistent with other randomized educational research trials published in PLOS ONE, which do not describe trial registration (e.g., <https://doi.org/10.1371/journal.pone.0214623> or <https://doi.org/10.1371/journal.pone.0214624>).  Institutional Ethical Committee approval is described in the Methods section under the subheading “IRB Statement”. In accordance with the Code of Federal Regulations 46.104(d), our study was conducted in an established educational setting and involved normal educational practices that were not likely to adversely impact students' opportunity to learn required educational content (see <https://www.ecfr.gov/current/title-45/subtitle-A/subchapter-A/part-46/subpart-A/section-46.104>). The randomized trial was deemed exempt in the spring of 2019 by Dr. Sharon Gieselmann, who was chair of our university’s IRB at the time. The exemption letter is attached. For further information, please contact the current chair of the IRB, Dr. Kate Schwartzkopf-Phifer. | We updated the subheading “IRB Statement” to “Ethics Statement” to better highlight this section.  We uploaded the IRB approval letters to the EMSS portal. |

## Comment: “As per the policy of journal, Authors are required to make all data underlying the findings described fully available, without restriction.”

| **Response** | **Changes made** |
| --- | --- |
| There are no restrictions on sharing the data. We have uploaded the minimal anonymized data set to a stable, public repository. | All data are available at the following web address: <https://osf.io/qw4x9/>. |

# Comments on Journal Requirements

## Comment: “Please ensure that your manuscript meets PLOS ONE's style requirements.”

| **Response** | **Changes made** |
| --- | --- |
| We have confirmed that all style requirements are met. | Figure names were changed to fit style requirements. |

## Comment: “Please provide additional details regarding participant consent.”

| **Response** | **Changes made** |
| --- | --- |
| We updated the informed consent description, including how it was obtained, witnessed, and stored. | “Ethics Statement” subsection of the Methods was updated. |

## Comment: “We note that you have indicated that data from this study are available upon request. PLOS only allows data to be available upon request if there are legal or ethical restrictions on sharing data publicly.”

| **Response** | **Changes made** |
| --- | --- |
| There are no restrictions on sharing the data. We have uploaded the minimal anonymized data set to a stable, public repository. | All data are available at the following web address: <https://osf.io/qw4x9/>. |

# Comments from Reviewer #1

## Rating: Unclear whether statistical analysis was appropriate. Data were not provided.

| **Response** | **Changes made** |
| --- | --- |
| We have uploaded the minimal anonymized data set to a stable, public repository. We hope that this will allow the reviewer to run a separate analysis and confirm our findings. | All data are available at the following web address: <https://osf.io/qw4x9/> |

## Comment: “The study entitled ‘Dependence of learning outcomes in flipped and lecture classrooms on review questions’ is well conducted and written. However, Discussion needs to be more elaborate as its the heart of the study. Add some relevant references and discuss your findings more extensively.”

| **Response** | **Changes made** |
| --- | --- |
| We agree that the discussion section could be more elaborate. We have expanded our discussion section to better relate our findings to previous work. | The Discussion section was substantially reorganized and rewritten. Subheadings were included to provide structure. Previously cited studies are discussed in further detail to note similarities and differences with our work. Additional citations were included when appropriate. |

# Comments from Reviewer #2

## Rating: Manuscript was not presented in an intelligible fashion and written in standard English.

| **Response** | **Changes made** |
| --- | --- |
| We believe the reviewer may have mistakenly selected this answer for the following reasons. 1) No specific errors were noted by Reviewer 2. 2) Reviewer 1 did not indicate any issues with grammar and commented that the manuscript was well written. 3) Both authors are native English speakers.  We will carefully review the manuscript to correct any unclear, incorrect, or ambiguous statements. | - |

## Comment: “I am very happy to review your manuscript. You showed the efficacy on the flipped cohort learning outcomes. It may seemed to be informative and helpful for the education. However, there are fatal problems. One of the biggest problem is that your results have had no power to make feel something new, "NOIES".”

| **Response** | **Changes made** |
| --- | --- |
| The reviewer described the findings of Study 2 but omitted Study 1. We agree that Study 2 is largely a conformational study that supports the findings of Study 1. Collectively, the results of Studies 1 & 2 are important to the field of education. The strong push for flipped classrooms is bolstered by studies that show improvements in learning outcomes in flipped class sections compared to traditional lecture classes. However, we believe that the flipped classroom setting is not what drives improvements in learning outcomes. Instead, learning outcomes improve as a function of repeated review, rather than class format. We believe that the focus on class format is misguided. While flipped classes can allow for repeated review, so can lecture classes. This is demonstrated in Study 1, where class format is varied and repeated review is held constant. In Study 2, we varied the amount of repeated review and observed significant differences in exam scores, reproducing the effects reported in other flipped class studies. Importantly, we also reported a difference in exam scores when we compared two flipped classroom sections: the flipped section with higher scores was exposed to additional review questions.  We are not sure what NOIES means. We are sorry that we cannot address this comment any further. We hope that the updated manuscript addresses the shortcomings. | The Results section was organized with subheadings to highlight the complementary nature of the two studies. The same subheadings were added to the Discussion section to connect the two sections.  The results of Study 2 includes additional text on the effect of repeated review on exam scores in: 1) two different flipped class sections and 2) two different lecture class sections. |
